# Use of Activity-Based Probes to Develop High Throughput Screening Assays That Can Be Performed in Complex Cell Extracts

**DOI:** 10.1371/journal.pone.0011985

**Published:** 2010-08-05

**Authors:** Edgar Deu, Zhimou Yang, Flora Wang, Michael Klemba, Matthew Bogyo

**Affiliations:** 1 Department of Pathology, Stanford School of Medicine, Stanford, California, United States of America; 2 Department of Biochemistry, Virginia Polytechnic Institute and State University, Blacksburg, Virginia, United States of America; 3 Department of Microbiology and Immunology, Stanford School of Medicine, Stanford, California, United States of America; The Scripps Research Institute, United States of America

## Abstract

**Background:**

High throughput screening (HTS) is one of the primary tools used to identify novel enzyme inhibitors. However, its applicability is generally restricted to targets that can either be expressed recombinantly or purified in large quantities.

**Methodology and Principal Findings:**

Here, we described a method to use activity-based probes (ABPs) to identify substrates that are sufficiently selective to allow HTS in complex biological samples. Because ABPs label their target enzymes through the formation of a permanent covalent bond, we can correlate labeling of target enzymes in a complex mixture with inhibition of turnover of a substrate in that same mixture. Thus, substrate specificity can be determined and substrates with sufficiently high selectivity for HTS can be identified. In this study, we demonstrate this method by using an ABP for dipeptidyl aminopeptidases to identify (Pro-Arg)_2_-Rhodamine as a specific substrate for DPAP1 in *Plasmodium falciparum* lysates and Cathepsin C in rat liver extracts. We then used this substrate to develop highly sensitive HTS assays (Z’>0.8) that are suitable for use in screening large collections of small molecules (i.e >300,000) for inhibitors of these proteases. Finally, we demonstrate that it is possible to use broad-spectrum ABPs to identify target-specific substrates.

**Conclusions:**

We believe that this approach will have value for many enzymatic systems where access to large amounts of active enzyme is problematic.

## Introduction

One of the most common techniques used by the pharmaceutical industry to identify novel drug leads is high throughput screening (HTS). This method allows inhibition effects of large numbers of compounds to be determined in a relative short period of time. HTS assays have traditionally been performed with either a recombinant form of the target enzyme or with purified native enzyme [1]. More recently, HTS has been performed using both cell-based and extract-based assays [2], [3]. While these types of assays avoid the need to express and purify a target enzyme, they often rely on genetically engineered reporter systems that tend to have a high rate of false positives. To get around this problem, it is possible to enhance the expression level of the targeted activity to reduce the background noise of the system [4]. Regardless, a specific inhibitor (often identified using recombinantly expressed enzyme) [1], [5] or a genetic knock-out of the target enzyme [4], [6] is needed to prove that the assay is target-specific. Therefore, in almost all cases, these assays have been developed for targets or systems that are amenable to genetic manipulation and/or protein engineering.

However, not all organisms are genetically tractable, and many enzymes cannot be purified or produced recombinantly in an active form. This is especially true for enzymes that are naturally expressed as zymogens and require posttranslational modification (proteolytic cleavage, phosphorylation, glycosylation, etc.) to become active, or those for which specific interactions with cellular components are required (protein-protein interaction, cofactors, etc.). Activity-based probes (ABPs) are ideally suited to assess binding and inhibition of target enzymes in the context of complex protein mixtures. Because they covalently modify the catalytic residue of the targeted enzyme, they can be used in competition assays to assess both potency and selectivity of compounds in intact cells, extracts and even whole organisms [7], [8]. However, the readout for such assays requires SDS-PAGE to measure residual target labeling by the probe. Therefore, this approach is not suitable for use in HTS. Alternatively, a recent study demonstrated the use of ABPs as reporters of enzyme activity for HTS. This study demonstrated that measuring changes in fluorescence anisotropy of the tag on an ABP as it binds its target can provide a sufficiently sensitive and quantitative readout of labeling to allow HTS [9]. Because labeling of the target is used as the readout of the assay, it is particularly valuable for enzymes for which suitable substrates have not been identified. However, this approach requires expressed or purified enzymes because the background of probe labeling in crude extracts is often high.

Alternatively, once a sufficiently selective substrate can be identified for a desired target enzyme, it is possible to directly measure its inhibition in complex mixtures. Here we demonstrate the use of ABPs to assess the selectivity of reporter substrates in crude cell extracts. We demonstrate that this approach facilitates the identification of substrates whose kinetics of turnover inhibition perfectly correlate with the kinetics of labeling of the target enzyme by the ABP. Such substrates can be deemed to be selective for the target enzyme and can therefore be use for HTS. In this study, we demonstrate the application of this method using an ABP that targets dipeptidyl aminopeptidases. Specifically, we use the probe in crude cell extracts from the human malaria parasite *Plasmodium falciparum* and in crude rat liver extracts to identify a highly selective substrate for dipeptidyl aminopeptidase 1 (DPAP1) and cathepsin C (Cat C). We then demonstrate that this substrate can be used to develop a highly sensitive and stable assay that is suitable for use in HTS with large libraries of small molecules.

## Results

We were initially interested in developing an assay that could be used in HTS to identify inhibitors of DPAP1, a protease that is expressed by the human malaria parasite *Plasmodium falciparum*. This enzyme is an essential cysteine protease involved in the final stages of hemoglobin degradation [10], [11]. DPAP1 is refractory to genetic disruption [11] and its inhibition blocks parasite growth both *in vitro* and *in vivo* [10], thus making it a potentially valuable anti-malaria drug target. We also chose to focus on this target because proteases are generally difficult to express in their active form as most of them are translated as inactive pro-enzymes. Furthermore, expression of *P. falciparum* proteins is especially challenging due to the A/T-rich nature of the genome of this organism. Additionally, DPAP1 is an ideal enzyme to demonstrate the value of our approach because we already identified a suitable ABP for this target [12], [13], we had specific information about its substrate specificity [10], [12], [14], and we had access to recombinantly expressed enzyme for comparison of our method with standard screening assays [14].

**Figure 1:**
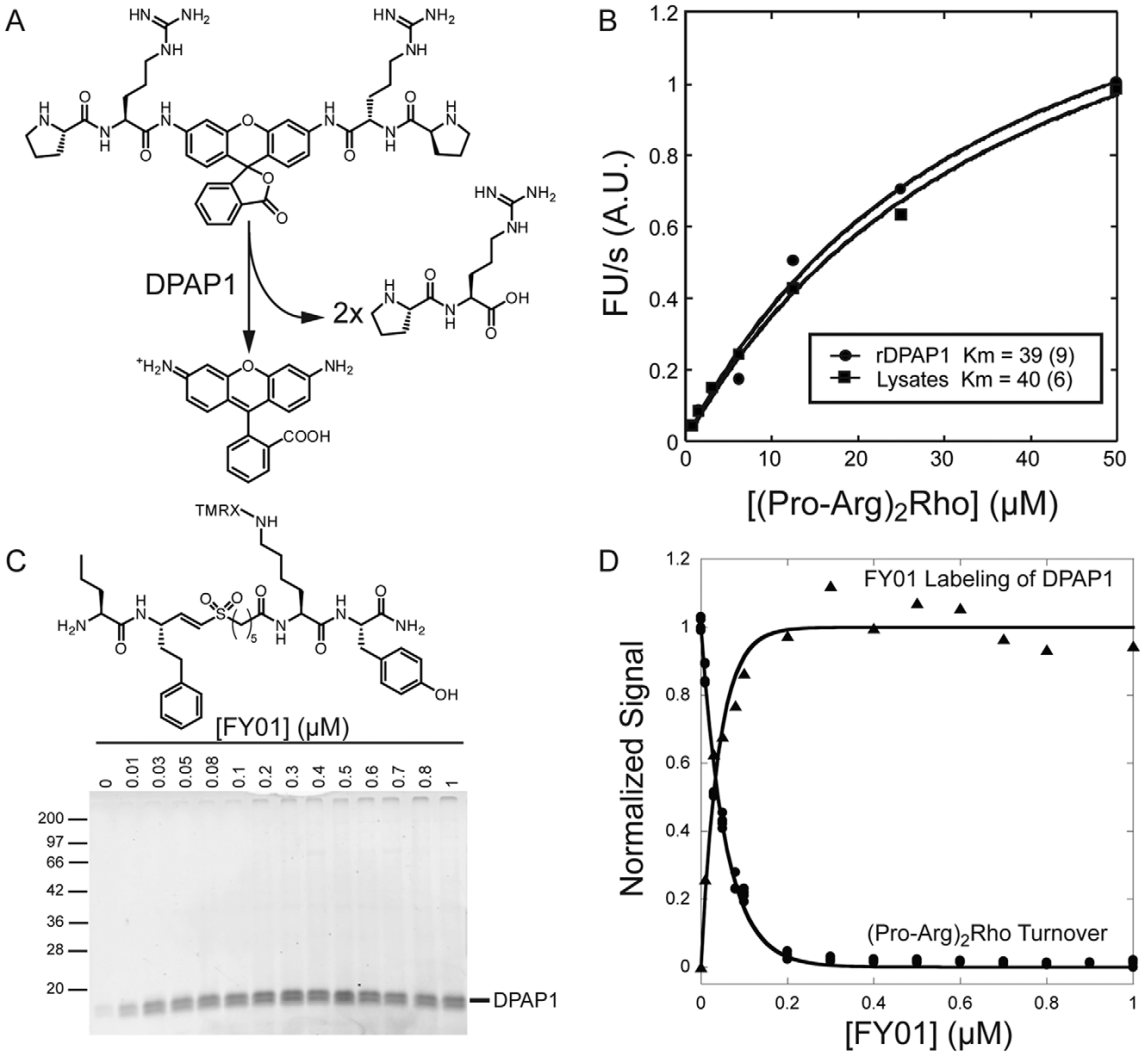
Use of an ABP to identify a DPAP1-selective substrate in parasite lysates. **A.** Structure and reaction mechanism of the (Pro-Arg)_2_-Rho substrate. **B.** Measurement of (Pro-Arg)_2_-Rho apparent *K*_m_ in trophozoite lysates (circles) and with recombinant DPAP1 (triangle). Turnover rates at increasing concentrations of substrate were fitted to a Michaelis-Menten equation as described in the methods section. **C.** Labeling of DPAP1 activity in parasite lysates with FY01. Trophozoite lysates were incubated for 1 h with increasing concentrations of FY01. Labeling was stopped by boiling the sample in SDS-PAGE loading buffer. DPAP1 activity was measured using a flatbed fluorescent scanner. **D.** DPAP1 labeling correlates with substrate turnover inhibition. An aliquot of the samples treated for 1 h with FY01 was diluted in assay buffer containing 10 µM of (Pro-Arg)_2_-Rho, and the initial turnover rate was measured in a 96-well plate (circles). This turnover rate is plotted with the labeling quantified in C.

DPAPs recognize the N-terminal amine of substrate proteins and are efficient at cleaving dipeptide fluorogenic substrates. Our SAR studies on DPAP1 indicated that proline at P2 (N-terminal amino acid) provides selectivity towards DPAP1 relative to other cysteine proteases in *P. falciparum* such as DPAP3 or the falcipains [10], [12]. Additionally, the screen of a positional scanning library of 7-amino-4-methylcoumarin (AMC) substrates identified Arg as a preferred residue at P1 [14]. Therefore, we synthesized the (Pro-Arg)_2_-Rho substrate to target DPAP1 in parasite lysates (Figure 1A). This substrate is cleaved twice by DPAP1 to produce free rhodamine.

We chose a rhodamine-based substrate because free rhodamine emits at a wavelength (523 nm) that is high enough to be free from most of the auto-fluorescence background of compounds in a diverse library of small molecules. Using more traditional protease fluorogenic substrates, such as AMC-substrates for example, which emit at a lower wavelength, will likely result in an increase in the rate of false negatives during HTS, since a significant portion of the molecules in a library will likely emit light below 500 nm. Also, a bidentate substrate is likely to be more specific since it needs to be cleaved twice in order to produce an optimal signal.

To begin to assess the utility of this substrate for measuring DPAP1 activity in complex protein mixtures, we measured its apparent *K*_m_ value in parasite extracts (Figure 1B). Importantly, we obtained an apparent *K*_m_ value (36 µM) that was within experimental error of the value measured using recombinant DPAP1, suggesting that this substrate functions similarly against the native and recombinant enzymes. To test whether (Pro-Arg)_2_-Rho is processed specifically by DPAP1, we treated parasite lysates for 1 h with increasing concentrations of FY01 - a fluorogenic ABP developed in our lab to target dipeptidyl aminopeptidases [12], [13] - and measured both DPAP1 labeling (Figure 1C) and substrate turnover (Figure 1D). Because FY01 only labels DPAP1 in trophozoite lysates (Figure 1C) it was straightforward to quantify labeling of the enzyme by SDS-PAGE. We found that the dose dependent labeling of DPAP1 by FY01 perfectly correlates with the extent of inhibition of (Pro-Arg)_2_-Rho turnover (Figure 1D) suggesting that the substrate is processed virtually exclusively by DPAP1.

**Figure 2:**
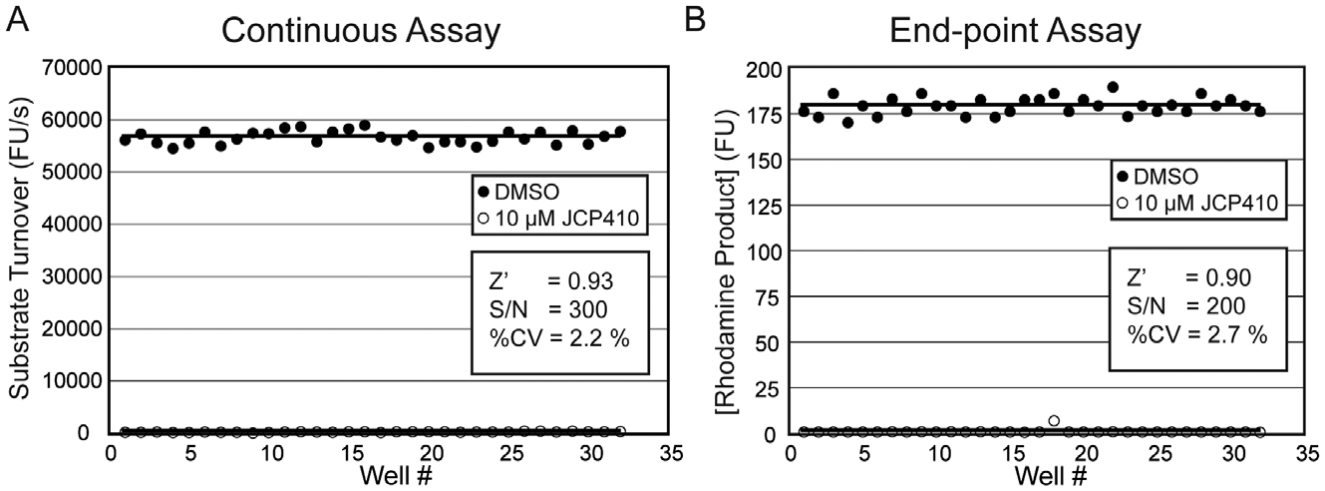
Development of a DPAP1-specific HTS assay. **A.** Continuous assay. The assay was carried out in 384-well plates using 1% of parasite lysates. Substrate turnover was continuously measured for 5 min. JCP410 (10 µM) was used as a positive inhibition control. Z’ factor, S/N, and % CV of the negative control are shown. **B.** End-point assay for HTS. The reaction described in A was quenched after 10 min by addition of 0.5 M acetic acid. The final concentration of rhodamine product was quantified by fluorescence.

Having demonstrated the selectivity of our substrate, we next wanted to determine if it could be used in a low-volume, 384-well plate assay. For the assay we continuously monitored substrate turnover for 5 minutes and then used the slope of the emission fluorescence over time as readout of enzyme activity. As a positive inhibition control, we used JCP410, a covalent inhibitor previously identified in our lab to be highly selective for DPAP1 [12]. Using this inhibitor and the continuous measurement of substrate processing, we could demonstrate that the assay has a signal-to-noise ratio (S/N) of 300 and an almost perfect Z’ factor (Figure 2A). Because end-point assays are generally preferred when screening large numbers of compounds, we adapted the assay into an end-point format by simply quenching the reaction after 10 min with acetic acid (data not shown). We evaluated this end-point assay in a fully automated format using a multi-channel peristaltic pump to dispense reagents into a 384-well plate, and a stackable plate reader. The end-point assay has a slightly lower S/N than the continuous assay, but the Z’-factor remains very high (0.9) (Figure 2B). Therefore, both the continuous and endpoint assays using the (Pro-Arg)_2_-Rho substrate are highly sensitive assays that are suitable for use in HTS with a large numbers of compounds. Furthermore, the use the 384 well format allows screening of large libraries using a relatively small amount of parasite lysates.

**Figure 3:**
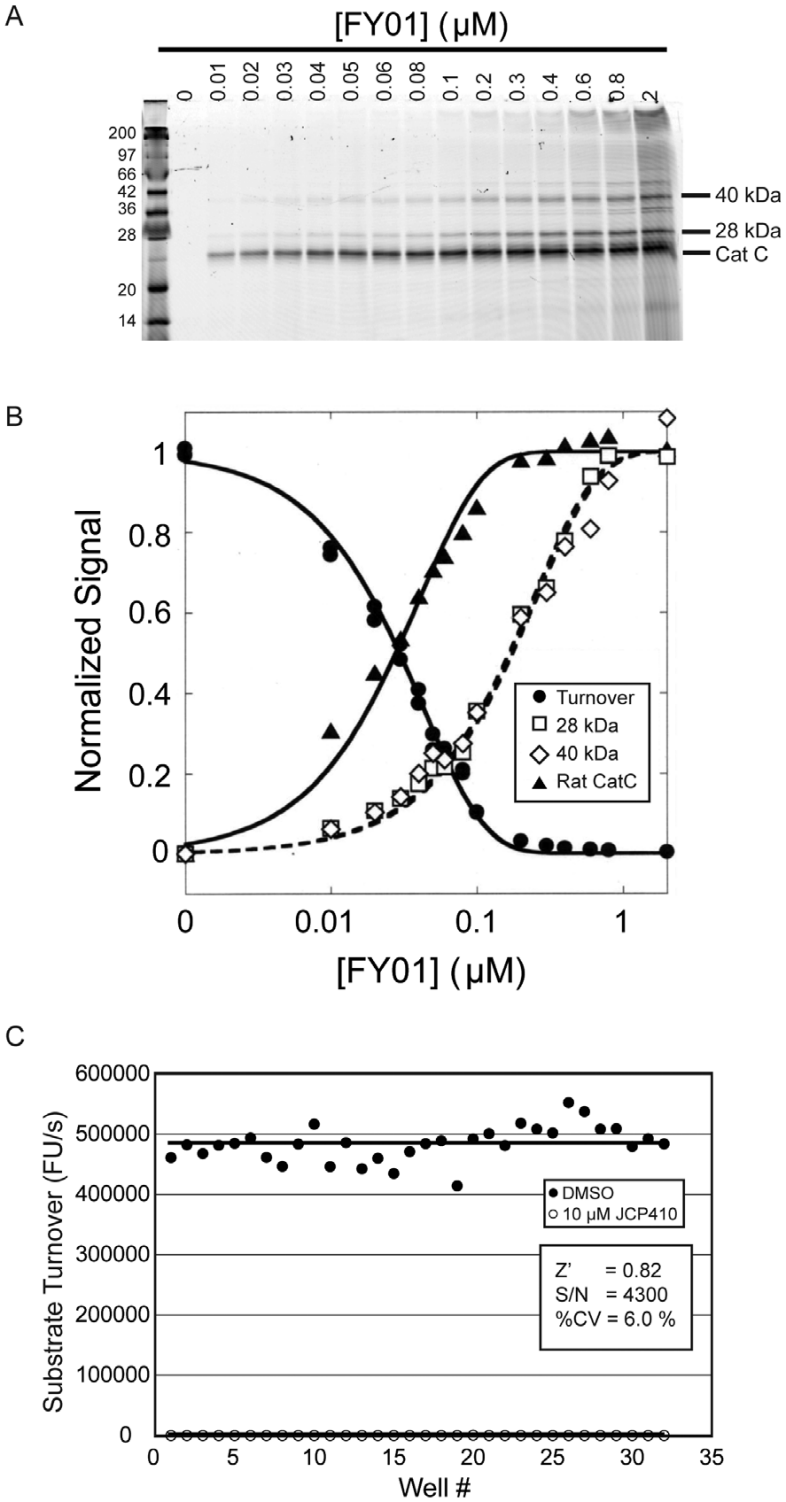
Cat C-specific fluorogenic assay in rat liver lysates. **A.** Labeling of Cat C with FY01. Rat liver extract extracts were treated with increasing concentrations of FY01 for 1 h and labeled proteins analyzed by SDS-PAGE followed by scanning of the gel using a flatbed laser scanner. The location of labeled Cat C is indicated. **B.** Inhibition of substrate turnover specifically correlates with Cat C labeling. The cleavage of (Pro-Arg)_2_-Rho substrate was measured prior to analysis of FY01 labeling shown in part A. Quantification of the indicated labeled proteins relative to DMSO control is shown. **C.** Cat C-specific HTS assay in rat liver extracts. Rat liver lysates were treated for 30 min with either DMSO or JCP410 (10 µM) followed by the addition of 10 µM of (Pro-Arg)_2_-Rho. The turnover rate was continuously measured for 5 min in a 384-well plate. Z’ factor, S/N, and % CV of the negative control are shown.

To demonstrate the general utility of our approach, we also applied the same methods to rat liver extracts. These samples are more complex than parasite lysates and contain a number of highly related papain fold cysteine proteases, in addition to the DPAP1 homolog Cat C. As expected, addition of FY01 to rat liver lysates resulted in labeling of multiple proteases targets (Figure 3A). However, only the labeling of rat Cat C (at 23 kDa) correlated with inhibition of (Pro-Arg)_2_-Rho turnover, suggesting that it is a highly selective substrate for Cat C in this system (Figure 3B). Furthermore, when the rat liver assay was converted to a 384-well plate format (20 µL reaction volume), we were also able to generate a high S/N and Z’ factor making it suitable for use in HTS (Figure 3C). Overall, these results demonstrate that our identified substrate can be used in multiple different extract systems and furthermore that ABPs that target multiple related enzymes can be used to identify specific substrates.

## Discussion

HTS remains one of the most powerful methods for identification of small molecule inhibitor leads. Before embarking on a large-scale screen, it is important to develop an assay that is sufficiently robust to allow the generation of meaningful results. For most enzymatic targets, HTS assays make use of highly purified proteins and well-defined substrates. This requirement for pure enzyme prevents the use of HTS for a large number of difficult to express proteins. For enzymes such as proteases, expression of mature, active enzymes is often challenging. This can be further compounded for organisms such as *P. falciparum* whose proteins typically do not express well in heterologous expression systems due to its high A/T-rich genome. Therefore, developing methods that allow rapid development of sensitive and robust assays that can be performed in crude protein extracts would enhance the number of targets that are accessible to HTS. In this study, we demonstrate that ABPs can be used to identify enzyme substrates that are sufficiently selective for use in HTS assays.

While the results presented here focus on protease targets, it should also be possible to use any of the ever-increasing number of ABPs (for review see [8], [15], [16]) to develop similar assay for other classes of enzymes. We demonstrate here that ABPs need not be selective for the target of interest but can bind many related targets. Since it is possible to correlate substrate processing with specific labeled proteins using an SDS-PAGE readout, the only limitation is the ability to resolve the probe-labeled proteins. Another possible limitation is access to a set of suitable substrates for a target of interest. However, because ABPs generally bind to target enzyme using similar mechanism as native substrates, they often serve as a starting points for design of substrate scaffolds. In addition, in the course of designing selective probes, a substantial amount of structure/activity information is usually generated. Thus, probes can be used to design substrates and vice versa. Even in situations where a perfectly selective substrate cannot be easily identified, it may be possible to use inhibitors to reduce the activity of other unwanted enzymes that contribute to substrate processing. Our approach is likely to be most successful for enzymatic activities that are highly abundant in an extract, either because the targeted enzyme is highly expressed or because it is the most efficient catalyst of substrate turnover. We anticipate that large and structurally diverse libraries of substrates can be used to identify specific substrates for enzymes that have low abundance or reduced catalytic efficiency.

Overall, the main advantage of our method is that it can be applied to any biological sample that can be obtained in sufficient quantity. A number of ABPs that target specific classes of enzymes have already been developed and can be used for this approach immediately. We believe that this approach will be useful to identify novel inhibitors for a variety of drug targets that cannot be expressed recombinantly, as well as to decrease the cost associated with HTS.

## Materials and Methods

### Synthesis of (Pro-Arg)_2_-Rho

The synthesis was based on previously described syntheses of similar rhodamine-based substrates [17], [18]. Rhodamine110 was reacted with Fmoc-Arg(Pbf)OH (6 eq) and pyridine (6 eq) for 24 h to yield [Fmoc-Arg(Pbf)]_2_-Rho. After purification by flash chromatography (1% methanol in dichloromethane), the 9-fluorenylmethylcarbamate (Fmoc) group was deprotected in a 1∶1 mixture of acetonitrile:diethylamine. HPLC purified [HN-Arg(Pbf)]_2_-Rho was reacted with Boc-Pro-OH (6 eq) in dry Dimethylformamide (DMF) in the presence of 2-(1H-benzotriazol-1-yl)-1,1,3,3-tetramethyluronium (HBTU; 6 eq), 1-hydroxybenzotriazole (HOBT; 6 eq), and Diisopropylethylamine (DIEA;18 eq) to yield [Boc-Pro-Arg(Pbf)]_2_-Rho. After deprotection of the *t*-butyloxycarbamate (BOC) in Frifluoracetic acid (TFA):H_2_O: tetrahydrofuran (THF) (95∶2.5∶2.5), (Pro-Arg)_2_-Rho was precipitated in cold ether and purified by high pressure liquid chromatography (HPLC). The substrate purity was determined by liquid chromatography/mass spectrometry (LC/MS) to be >95% pure.

### Cell lysates preparation

D10 *P. falciparum* parasites were cultured synchronously as described in [10]. Parasites were harvested at trophozoite stage (∼34 h post RBC invasion) by lysing the red blood cell (RBC) membrane with 0.15% saponin in phosphate buffered saline (PBS) and spinning down the parasite pellet. Those were lysed in 2 volumes of 1% nonidet P40 in PBS for 1 h in ice. The soluble fraction of the lysates was store at −80°C. Rat liver extracts were obtained as described in [19].

### Labeling of cysteine protease activity with ABPs

Extracts from *P. falciparum* or rat livers (purchased from Rockland Immunochemicals, Gilbertsville, PA) were diluted 10-fold in assay buffer (50 mM sodium acetate pH 5.5, 5 mM MgCl_2_, and 5 mM dithiothreitol (DTT)) and treated for 1 h with FY01 at room temperature. No APLAC approval was required to obtain rat liver extracts. Samples were boiled in SDS-loading buffer and run in an SDS-PAGE gel. Labeled proteins were detected in a 9410 Typhoon flatbed scanner (Ammersham Bioscience, GE Healthcare). The identity of the different bands in the gels for parasite and liver lysates has been described in [12] and [13], respectively.

### Continous fluorogenic assays

All assay were performed in assay buffer with 10 µM of (Pro-Arg)_2_-Rho substrate in 96- or 384-well plates (100 or 20 µL reaction volume, respectively), depending on whether the assay was used to validate the substrate as a target-specific substrate, or to test the assay for HTS purposes, respectively. The reaction was run with 1% of parasite lysates or with 0.1% of rat liver extract. Substrate turnover was measured for 5 min at 523 nm ( λ_excitation_ = 492 nm) in a Spectramax M5 plate-reader (Molecular Devices). Recombinant DPAP1 was prepared as described in [14].

### End-point assay for DPAP1

This assay was performed in 384-well plates. The compound addition, reagent dispensing and fluorescent readout of this end-point assay was fully automated. The reagents were added using a stackable dispenser equipped with a multichannel peristaltic pump (Matrix WellMate Bulk Dispenser). 10 µL of 2% trophozoite lysates in assay buffer were added to 10 µL of 20 µM (Pro-Arg)_2_-Rho in assay buffer. The plate was spin for 10 s to ensure that all the reaction volume was at the bottom of the well. After 10 min, the reaction was stopped by adding 20 µL of 0.5 M acetic acid. Fluorescence was measured at 530 nm (λ_excitation_ = 485 nm) with an Analyst AD stackable plate-reader (Molecular Devices).

### Statistic evaluation of the HTS assays

As a positive inhibition control, we used 10 µM of JCP410, which is a known covalent inhibitor of DPAP1 [12] and Cat C [13]. The inhibitor was added to parasite lysates at the same time as the substrate, or to rat liver extracts 30 min prior to the addition of the substrate. The Z’ factor and coefficient of variation (% CV) values were evaluated according to eqs 1 and 2, respectively.



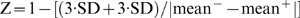
(1)

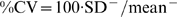
(2)where the positive and negative superindexes refer to the positive (JCP410) and negative (DMSO) inhibition controls (SD  =  standard deviation).
